# Feelings of being a second victim among Spanish midwives and obstetricians

**DOI:** 10.1002/nop2.1249

**Published:** 2022-05-28

**Authors:** Irene Santana‐Domínguez, Héctor González‐De La Torre, José Verdú‐Soriano, Miriam Berenguer‐Pérez, Juan José Suárez‐Sánchez, Alicia Martín‐Martínez

**Affiliations:** ^1^ University of Las Palmas de Gran Canaria (ULPGC) Las Palmas de Gran Canaria‐Canary Islands Spain; ^2^ Canary Health Service. Obstetrics and Gynaecology Department Gran Canaria Maternal and Infant University Hospital Complex Canary Islands Spain; ^3^ Research Unit of Insular Maternal and Child University Hospital Complex of Gran Canaria, Canary Health Service Las Palmas de Gran Canaria‐Canary Islands Spain; ^4^ University of La Laguna (ULL)‐Nursing Unit La Palma Tenerife‐Canary Islands Spain; ^5^ Department of Community Nursing, Preventive Medicine, Public Health and History of Science, Faculty of Health Sciences University of Alicante (UA) Alicante Spain; ^6^ Canary Health Service, Direction of Primary Care Gran Canaria Canary Islands Spain

**Keywords:** adverse events, midwives, obstetricians, second victim

## Abstract

**Aim:**

The aim of this study was to determine the prevalence of feelings of being a second victim among midwives and obstetricians in Spain and to explore possible differences between the two professions.

**Design:**

Cross‐sectional descriptive‐analytical observational study.

**Methods:**

An online survey collecting several variables was administered throughout the Spanish territory. Spanish version of the Second Victim Experience and Support Tool (SVEST) was used. The data collection period was from May to December 2020.

**Results:**

A total sample of 719 obstetricians and midwives were studied. There were significant differences between the two groups with respect to seven dimensions of SVEST: greater feelings of being a second victim among obstetricians in the dimensions physical distress/*p* ≤ .001, non‐work‐related support/*p* ≤ .001 and absenteeism/*p* ≤ .001 and greater feelings of being a second victim among midwives in the dimensions psychological distress/*p* ≤ .001, supervisor support/*p* = .011, professional self‐efficacy/*p* ≤ .001 and intention to change jobs/*p* ≤ .001.

What does this paper contribute to the wider global clinical community?

- Professionals who work in obstetric care and midwifery (midwives and obstetricians) suffer from a high prevalence of feelings of second victim. These feelings may be higher for midwives.
- The feeling of second victims influences not only the mental health of the professionals who suffer from it, but also affects other important areas such as the intention to turnover or to leave the profession.
- It is necessary to design specific support programmes for professionals involved in the phenomenon of second victims. These programmes should be designed taking into account the possible differences that may exist depending on the type of professional involved. Midwifery supervisors should have special training in this area.

## 1 INTRODUCTION

The clinical variability, the complexity of the care processes and the inherent degree of error associated with any human activity make the occurrence of unforeseen healthcare‐related adverse events inevitable, which implies an evident risk for patients (Coughlan et al., 2017). These events not only harm patients but also negatively affect health professionals, who, after having been involved in this type of incident, can become what are called “second victims” (Busch et al., 2020; Marran, 2019).

Although different definitions have been proposed for the term “second victim” since its introduction in 2000 (Wu, 2000), it may be defined as “doctors, nurses or other health professionals who have committed errors related to patient care and that have experienced psychological effects as a result” (Cabilan & Kynoch, 2017; Marran, 2019). These psychological effects are very diverse and broad, ranging from mild‐to‐very severe symptoms (Busch et al., 2020; Chan et al., 2017). Therefore, and due to the repercussions of this phenomenon, the study of the second victim phenomenon has boomed in recent years (Busch et al., 2020). In addition to the damage to health professionals, this phenomenon causes a significant economic and reputational impact on health systems and their institutions (Marran, 2019; White & Delacroix, 2020). Additionally, some studies relate the experience of second victim with burnout and leaving the profession (Burlison et al., 2021; Sheen et al., 2015).

Overall, the consequences of errors in patients are usually mild, but a percentage of these errors can cause permanent and serious injury and even lead to the death of the patient (Haukland et al., 2019). This is especially worrying in the realm of obstetric‐maternal care (Pettker, 2017).

Obstetric‐maternal care is a very sensitive healthcare area (Coughlan et al., 2017; Healy et al., 2016; Pettker, 2017). Society does not willingly accept the occurrence of errors associated with the health care of women during pregnancy and childbirth, since it assumes that in these cases; there is no margin for error and that the obstetric‐maternal outcomes should always be good, but this is clearly not the case (Coughlan et al., 2017; McDaniel & Morris, 2020).

Health professionals in the obstetric field, midwives and obstetricians are subject to high levels of tension and stress and are very susceptible to becoming second victims (Schrøder, Jørgensen, et al., 2016b; Schrøder, Larsen, et al., 2016). This is because sometimes an adverse event can lead to a traumatic delivery, a maternal–foetal death or a serious medical error related to perinatal care (Rivera‐Chiauzzi et al., 2021; Schrøder, Jørgensen, et al., 2016b; Schrøder, Larsen, et al., 2016; Wahlberg et al., 2019). A high percentage of midwives and obstetricians will experience serious obstetric events in their professional development that can affect them, sometimes severely (Coughlan et al., 2017; Pettker, 2017; Sheen et al., 2015).

This topic has been studied in obstetric professionals in some countries, both among midwives (Kerkman et al., 2019) (Buhlmann et al., 2021) and obstetric physicians (Baas et al., 2018; Torbenson et al., 2021), but there are still few studies investigating in depth the possible differences with respect to this phenomenon between the two professions (Rivera‐Chiauzzi et al., 2021; Schrøder et al., 2019; Wahlberg et al., 2017). Although both perform their professional work in the same health field, often working as a team, they are different professions and consequently have different connotations (Coates et al., 2021).

In Spain, obstetric care is carried out by midwives and obstetricians. Midwives are nurses with a 4‐year university degree who, also, have a 2‐year speciality and take care of women during pregnancy, childbirth and puerperium while these processes develop without problems. Obstetricians (a speciality after medical degree) develop their action in those pathological conditions or with complications related to these stages. Although some approaches have been undertaken about second victims (Carrillo et al., 2016; Mira et al., 2015) in Spain, the study of this phenomenon is scarce, and there are still no specific data on the prevalence and other aspects related to the phenomenon among Spanish health professionals working in the field of obstetrics. In addition, there are no studies that explore and compare the possible differences between both categories that provide care in the same obstetric area.

So, in order to provide evidence in this topic, two research questions were formulated as follows: What is the prevalence of the feeling of second victim among midwives and obstetricians in Spain? And are there differences in terms of physical, psychological or other consequences between both disciplines?

Therefore, according to those research questions, the objectives of the present study were to determine the prevalence of the feeling of second victim among midwives and obstetricians in Spain and to explore possible differences between the two professions with respect to the physical, psychological and other consequences of this phenomenon in these professionals.

## 2 METHODS

### 2.1 Design

A cross‐sectional observational study with an analytical component was conducted. The study is reported as per the Reporting of Observational Studies in Epidemiology Guidelines (STROBE) (Appendix S1).

### 2.2 Study population

Midwives and obstetricians working throughout the Spanish territory. The inclusion criteria were as follows: being a physician specializing in obstetrics and gynaecology or midwife and working in direct women’s care.

### 2.3 Sampling and data collection

Non‐probabilistic convenience sampling was used to select the sample. The research team contacted different Spanish professional associations by phone/email (Spanish Society of Gynaecology and Obstetrics‐SEGO, Federation of Midwifery Associations of Spain‐FAME, Spanish Association of Midwives‐AEM and the Union of Midwives of Spain‐SIMAES, for their acronyms in Spanish) with the objective of disseminating it among members the opportunity to participate in the study.

Professionals who wished to participate were sent a link via email, through which they accessed an online questionnaire on a secure online platform (*Google Forms®*) following the recommendations of the CHERRIES guidelines (Eysenbach, 2004). A total of 957 questionnaires were sent out. The data collection period lasted from 15 May to 31 December 2020 (Appendix S2).

Accordingly with the process, a priori sample size was not calculated as we did not have any expectations on the response rate; nevertheless, a post hoc precision for prevalence of the feeling of being a second victim, in general and among disciplines, was calculated, considering a 95% confidence level. In addition, a post hoc statistical power of the sample was calculated for the difference in the total mean score on the SVEST‐E between both groups of professionals.

### 2.4 Variables and data collection instrument

The online questionnaire consisted of two parts. The first part was created ad hoc for collection of the study variables: gender, age, marital status, professional category (obstetrician or midwife), the highest level of education reached, years of professional experience, type of work centre (public or private), type of unit/department (hospital/specialized care or primary care), region of the country, knowledge of the term “second victim” (null, medium or high), existence of support programmes for second victims in the workplace (yes/no/do not know), feeling of having been a second victim at some point (yes/no) and approximate time of the event that caused the feeling of second victim.

The second part included the SVEST‐E (Santana‐Domínguez et al., 2021) (Santana‐Domínguez et al., 2022), the Spanish version of the Second Victim Experience and Support Tool (SVEST) (Burlison et al., 2017). This instrument functions as a survey questionnaire that specifically measures the second victim phenomenon and has been validated and used in numerous countries and settings (Brunelli et al., 2018; Chen et al., 2019; Kim et al., 2020; Knudsen et al., 2021; Mok et al., 2020; Scarpis et al., 2021). The SVEST‐E retains the same items as the original version of the SVEST, although it makes changes in the organization of the dimensions (Santana‐Domínguez et al., 2022). To be able to make comparisons with other studies, the original structure of the original SVEST was maintained for analysis in this study.

The original SVEST considers seven dimensions: Psychological distress (4 items), Physical distress (4 items), Colleague support (4 items), Supervisor support (4 items), Institutional support (3 items), Non‐work‐related support (2 items) and Personal self‐efficacy (4 items). The instrument also assesses two outcome variables–dimensions: Intention to change jobs (2 items) and Absenteeism (2 items). It also provides a section with seven items as response options for second victims to report their preferred forms of support desired from their organizations (Burlison et al., 2017).

The SVEST uses a 5‐point Likert scale with scores ranging from 1 (“strongly disagree”) to 5 (“strongly agree”). Higher scores are associated with a greater experience and feeling of second victim in the professional (Burlison et al., 2017). The sum of the scores of the nine dimensions allows obtaining a total score about the feeling of second victim of the professional, in addition to the score for each dimension (Burlison et al., 2017).

The answers on the section about desired forms of support are also scored on a Likert scale of 1–5, where 1 represents “strongly do not desire” and 5 “strongly desire” and allows obtaining detailed and concrete information on possible forms of support desired by professionals involved as second victims. A response of 4 or 5 is representative of a desirable support option, while a response of 1 or 2 is indicative that the support option is undesirable (Burlison et al., 2017; Mok et al., 2020). The Cronbach’s alpha coefficient was 0.834 for our study.

### 2.5 Data analysis

A descriptive analysis of the variables was performed with the statistical program IBM© SPSS Statistics v.24.0.

In the first phase, a descriptive analysis of the variables was performed, with the categorical variables expressed as percentages and frequencies and quantitative variables as means, standard deviations and minimum and maximum values.

In a second phase, an inferential analysis was performed. To establish whether there were differences between the groups, the Pearson chi‐square test was used for categorical variables. To test the hypothesis of normality in the distribution of the data, the Kolmogorov–Smirnov test was used. If the distribution was normal, Student’s *t*‐test was applied for the comparison of means; if the data did not present a normal distribution, then the nonparametric Mann–Whitney test was applied, adopting a significance level of α = 0.05.

The effect size was calculated, as defined by Cohen (Cohen, 1988), as the difference between the mean scores of the study groups divided by the combined standard deviation of the two groups. Effect sizes between 0.2 and 0.5 were considered “small,” between 0.5 and 0.8 as “moderate” and above 0.8 as “large.”

The degree of agreement of the participants with each of the dimensions was calculated as the percentage of participants who answered “agree” or “strongly agree” to each item (Burlison et al., 2017; Mok et al., 2020).

### 2.6 Ethics considerations

The study was evaluated and approved by the Research Ethics Committee/Drug Research Ethics Committee of Dr. Negrin University Hospital of Gran Canaria (Code n. 2020–140‐1). The professionals who agreed to participate did so voluntarily and were given all the information on the objectives of the project in its entirety. They gave their informed consent by voluntarily accessing the online questionnaire and completing it. Confidentiality and anonymity were ensured in all phases of the investigation. For the analysis of the data, a blinded matrix was used where no identifiable participant data appeared.

## 3 RESULTS

### 3.1 Sociodemographic characteristics of the sample

The final sample was composed of a total of 719 professionals from a total of 19 Spanish regions (Response rate 75.13%). The region with the highest participation was the Community of the Canary Islands with 111 participants (15.4%) and Andalusia (93/12.9%) and the one with the lowest participation was Ceuta (13/1.8%). A total of 330 (45.9%) professionals were obstetricians, and 389 (54.1%) were midwives. In both cases, the majority were women (473/65.8%). The mean age of the participants was 43.11 (*SD* = 10.54) years [minimum‐maximum age: 25–71 years], with an average professional experience of 14.57 (*SD* = 10.46) years [minimum–maximum professional experience: 0–45 years]. Table 1 shows the frequencies and percentages of the study variables for each of the groups (obstetricians and midwives).

**TABLE 1:** Sociodemographic characteristics of the sample and variables

Variable	Obstetricians *N* = 330	Midwives *N* = 389	Pearson *X* ^2^
	*N* (%)	*N* (%)	
Gender			*p* = .080
Female	206 (62.4)	267 (68.6)	
Male	124 (37.6)	122 (31.4)	
Feeling of a second victim			*p* = .934
Yes	206 (62.4)	244 (62.7)	
No	124 (37.6)	145 (37.3)	
Civil Status			*p* = .001*
Married	152 (46.1)	141 (36.2)	
Single	90 (27.3)	167 (42.9)	
Divorced	32 (9.7)	33 (8.5)	
Separated	34 (10.3)	31 (8.0)	
Widowed	11 (3.3)	7 (1.8)	
Other	11 (3.3)	10 (2.6)	
Maximum educational level reached			*p* ≤ .001*
University	120 (36.4)	146 (37.5)	
Expert	37 (11.2)	102 (26.2)	
Master	97 (29.4)	100 (25.7)	
PhD	59 (17.9)	35 (9.0)	
Other	17 (5.2)	6 (1.5)	
Type of centre			*p* = .003*
Public centre	262 (79.4)	313 (80.5)	
Private centre	60 (18.2)	75 (19.3)	
Other centre types	8 (2.4)	1 (0.3)	
Department			*p* = .007*
Hospital	249 (75.5)	258 (66.3)	
Health centre	81 (24.5)	131 (33.7)	
Knowledge of the term “second victim”^a^			*p* = .005*
Null	133 (40.3)	203 (52.2)	
Medium	156 (47.3)	143 (36.8)	
High	41 (12.4)	43 (11.1)	
Existence of support programmes for second victims in the workplace			*p* = .003*
Yes	60 (18.2)	38 (9.8)	
No	177 (53.6)	218 (56.0)	
Does not know	93 (28.2)	133 (34.2)	
If you felt like a second victim, did you report the event?			*p* = .698
Yes	107 (32.4)	117 (30.1)	
No	99 (30.0)	127 (32.6)	
Time elapsed since the event that caused the feeling of being a second victim			*p* = .540
Less than a year	58 (17.6)	80 (20.6)	
Between 1 year and 2 years	26 (7.9)	36 (9.2)	
More than two years	109 (33.0)	119 (30.6)	

Variable	*M* (SD)	*M* (SD)	Mann–Whitney U
Age	46.45 (9.98)	40.28 (10.17)	*p* ≤ .001**
Years of professional experience	16.28 (10.05)	13.13 (10.60)	*p* ≤ .001**

Abbreviations: *M*, mean; SD, standard deviation. *Statistically significant Pearson *X* ^*2*^. **Statistically significant Mann–Whitney U Test.

^a^ Null = I have never heard of the term “second victim”/Medium = I have heard of the term “second victim” and know its meaning previously/High = I have extensive knowledge on the term “second victim.”

### 3.2 Prevalence of the feeling of being a second victim

The general prevalence of the feeling as a second victim was 62.6% (CI95% = 58.9%–66.1%). A post hoc precision analysis for this sample size revealed a 3.5% precision with a 95% confidence level. As you can see on Table 1, the prevalence for obstetricians was 62.4% (CI95% = 57.0%–67.7%) and 62.7% (CI95% = 57.7%–67.5%) for midwives, without statistical significance between groups.

### 3.3 SVEST‐E score

The mean score on the SVEST‐E questionnaire for the total sample was 3.07 (*SD* = 0.51). The highest scores were obtained on the Psychological distress dimension (3.68/*SD* = 1.01) and the lowest on the Absenteeism dimension (2.49/*SD* = 1.24). The item with the highest score was “1.3‐My experiences have made me feel sad,” with a score of 4.03 (*SD* = 1.12), and the item with the lowest score was “6.2‐The love of my closest friends and family helps me get through these events,” with a score of 1.56 (*SD* = 0.77).

Table 2 shows the floor percentage, the ceiling percentage, the mean and the standard deviation of each of the items included in the SVEST‐E questionnaire for the total sample, as well as the mean scores on the nine dimensions. The percentage of agreement of the participants with each of the dimensions was also collected.

**TABLE 2:** SVEST scores and percentage of agreement in the total sample (*n* = 719)

SVEST‐E items and dimensions	Floor Strongly disagree^a^	Ceiling Strongly agree^a^	*M* (*SD*)	Agreement^b^
	*n* (%)	*n* (%)		*n* (%)
Dimension 1—Psychological distress			3.68 (1.01)	361 (50.20)
1.1 I have experienced embarrassment from these instances.	57 (7.9)	205 (28.5)	3.59 (1.26)	
1.2 My involvement in these types of instances has made me fearful of future occurrences.	32 (4.5)	230 (32.0)	3.82 (1.15)	
1.3 My experiences have made me feel miserable.	15 (2.1)	316 (43.9)	4.03 (1.12)	
1.4 I feel deep remorse for my past involvements in these types of events.	86 (12)	193 (26.8)	3.29 (1.39)	
Dimension 2—Physical distress			3.16 (1.10)	247 (35.35)
2.1 The mental weight of my experience is exhausting.	38 (5.3)	185 (25.7)	3.63 (1.19)	
2.2. My experience with these occurrences can make it hard to sleep regularly.	76 (10.6)	138 (19.2)	3.32 (1.29)	
2.3 The stress from these situations has made me feel queasy or nauseous.	160 (22.3)	68 (9.5)	2.76 (1.32)	
2.4. Thinking about these situations can make it difficult to have an appetite.	127 (17.7)	82 (11.4)	2.92 (1.30)	
Dimension 3—Colleague support			2.74 (0.54)	6 (0.83)
3.1 I appreciate my co‐workers' attempts to console me, but their efforts can come at the wrong time.	32 (4.5)	215 (29.9)	3.70 (1.19)	
3.2 Discussing what happened with my colleagues provides me with a sense of relief.^R^	313 (43.5)	9 (1.3)	1.84 (0.97)	
3.3 My colleagues can be indifferent to the impact these situations have had on me.	59 (8.2)	213 (29.6)	3.56 (1.30)	
3.4 My colleagues help me feel that I am still a good healthcare provider despite any mistakes I have made.^R^	300 (41.7)	14 (1.9)	1.86 (0.97)	
Dimension 4—Supervisor support			2.90 (0.93)	113 (15.72)
4.1. I feel that my supervisor treats me appropriately after these occasions.^R^	91 (12.7)	92 (12.8)	3.05 (1.25)	
4.2 My supervisor’s responses are fair.^R^	85 (11.8)	94 (13.1)	3.15 (1.24)	
4.3 My supervisor blames individuals.	91 (26.6)	20 (2.8)	2.29 (1.08)	
4.4 I feel that my supervisor evaluates these situations in a manner that considers the complexity of patient care practices.^R^	80 (11.1)	89 (12.4)	3.12 (1.23)	
Dimension 5—Institutional support			3.58 (0.99)	342 (47.57)
5.1 My organization understands that those involved may need help to process and resolve any effects they may have on care providers.^R^	40 (5.6)	173 (24.1)	3.63 (1.17)	
5.2 My organization offers a variety of resources to help me get over the effects of involvement with these instances.^R^	44 (6.1)	206 (28.7)	3.76 (1.16)	
5.3. The concept of concern for the well‐being of those involved in these situations is not strong at my organization.	82 (11.4)	160 (22.3)	3.36 (1.32)	
Dimension 6—Non‐work‐related support			1.64 (0.78)	21 (2.92)
6.1 I look to close friends and family for emotional support after one of these situations happens.^R^	354 (49.2)	10 (1.4)	1.72 (0.90)	
6.2 The love from my closest friends and family helps me get over these occurrences.^R^	409 (56.9)	4 (0.6)	1.56 (0.77)	
Dimension 7—Professional self‐efficacy			3.57 (0.91)	313 (43.53)
7.1. Following my involvement I experienced feelings of inadequacy about my patient care abilities.	33 (4.6)	124 (17.2)	3.40 (1.16)	
7.2. My experience makes me wonder if I am not really a good healthcare provider.	43 (6.0)	191 (26.6)	3.52 (1.27)	
7.3 After my experience, I became afraid to attempt difficult or high‐risk procedures.	35 (4.9)	262 (36.4)	3.85 (1.19)	
7.4 These situations do not make me question my professional abilities.^R^	59 (8.2)	147 (20.4)	3.50 (1.22)	
8 ‐ Outcome variable 1—Intention to change jobs			3.01 (1.31)	304 (42.28)
8.1. My experience with these events has led to a desire to take a position outside of patient care.	125 (17.4)	96 (13.4)	3.03 (1.34)	
8.2. Sometimes the stress from being involved with these situations makes me want to quit my job.	139 (19.3)	112 (15.6)	3.00 (1.39)	
9 ‐ Outcome variable 2—Absenteeism			2.49 (1.24)	182 (25.31)
9.1. My experience with an adverse patient event or medical error has resulted in me taking a mental health day.	194 (27.0)	52 (7.2)	2.52 (1.29)	
9.2. I have taken time off after one of these instances occurs.	201 (28.0)	48 (6.7)	2.45 (1.27)	

Abbreviations: *M*, mean; *SD*, standard deviation. R = the ratings of statements written in negative terms are reversed.

^a^ Only upper (ceiling) or lower (floor) responses are displayed per item. Ceiling responses refer to “Strongly agree” and Floor responses refer to “Strongly disagree.”

^b^ Percentage of agreement of the different dimensions was represented by the percentage of respondents with an overall mean dimension score of ≥4.0. (Scores Agree = 4 or Strongly agree = 5).

### 3.4 Inferential analysis

Significant differences were found between the obstetrician and midwife groups with respect to age and years of professional experience as well as with respect to marital status, the highest level of education reached, type of centre and department, knowledge of the term “second victim” and existence of a support programme at the workplace (Table 1). No differences were found in regard to the feeling of having been a second victim at some point (*X* ^2^ *p* = .934) or with respect to the approximate time of the event that caused this feeling (*X* ^2^ *p* = .540). Participants from both groups informed their institutions of the event that had caused the feeling of second victim at a similar rate (*X* ^2^ *p* = .698) (Table 1).

The total mean score on the SVEST‐E was 3.01(*SD* = 0.52) for obstetricians and 3.13 (*SD* = 0.49) for midwives, and this result was significantly different (Mann–Whitney U *p* = .003; Cohen’s effect size = 0.23). A post hoc power analysis for this comparison was made considering equal variances and a difference of 0.12 to detect with a common *SD* of 0.5 (0.52 for obstetricians and 0.49 for midwives). Then, a statistical power of 89.5% was achieved.

Table 3 shows the means and standard deviations by items and dimensions for the obstetrician and midwife groups, as well as the *p*‐values obtained and the effect size. There were significant differences between the groups with respect to seven of the nine dimensions: In three dimensions, there was greater feeling of second victim among obstetricians (Physical distress/*p* ≤ .001, Non‐work‐related support/*p* ≤ .001 and Absenteeism/*p* ≤ .001), and in four, there was greater feeling of second victim among midwives (Psychological distress/*p* ≤ .001, Supervisor support/*p* = .011, Professional self‐efficacy/*p* ≤ .001 and Intention to change jobs/*p* ≤ .001).

**TABLE 3:** SVEST scores for the midwife and obstetrician groups

	Obstetricians *N* = 330	Midwives *N* = 389	Mann–Whitney U	Cohen`s effect size^a^
	*M* (*SD*)	*M* (*SD*)		
Dimension 1—Psychological distress	3.56 (1.02)	3.77 (0.99)	≤0.001**	0.21
1.1 I have experienced embarrassment from these instances.	3.47 (1.32)	3.69 (1.21)	0.035**	0.17
1.2. My involvement in these types of instances has made me fearful of future occurrences.	3.73 (1.22)	3.89 (1.09)	0.152	0.14
1.3 My experiences have made me feel miserable.	4.04 (1.12)	4.02 (1.12)	0.745	0.02
1.4 I feel deep remorse for my past involvements in these types of events.	3.02 (1.43)	3.51 (1.31)	≤0.001**	0.36
Dimension 2—Physical distress	3.19 (1.08)	3.13 (1.12)	≤0.001**	0.05
2.1 The mental weight of my experience is exhausting.	3.62 (1.15)	3.64 (1.22)	0.562	0.02
2.2 My experience with these occurrences can make it hard to sleep regularly.	3.35 (1.26)	3.30 (1.32)	0.664	0.04
2.3 The stress from these situations has made me feel queasy or nauseous.	2.85 (1.31)	2.68 (1.32)	0.077	0.13
2.4 Thinking about these situations can make it difficult to have an appetite.	2.94 (1.30)	2.90 (1.31)	0.712	0.03
Dimension 3—Colleague support	2.76 (0.57)	2.72 (0.51)	0.337	0.07
3.1 I appreciate my co‐workers' attempts to console me, but their efforts can come at the wrong time.	3.67 (1.23)	3.72 (1.16)	0.699	0.04
3.2 Discussing what happened with my colleagues provides me with a sense of relief.^R^	1.92 (1.01)	1.77 (0.92)	0.051	0.15
3.3 My colleagues can be indifferent to the impact these situations have had on me.	3.53 (1.31)	3.59 (1.29)	0.532	0.05
3.4 My colleagues help me feel that I am still a good healthcare provider despite any mistakes I have made.^R^	1.94 (1.03)	1.80 (0.91)	0.122	0.14
Dimension 4—Supervisor support	2.77 (0.95)	3.02 (0.90)	0.011**	0.27
4.1 I feel that my supervisor treats me appropriately after these occasions.^R^	2.88 (1.26)	3.19 (1.22)	≤0.001**	0.25
4.2 My supervisor’s responses are fair.^R^	2.99 (1.26)	3.29 (1.21)	≤0.001**	0.24
4.3 My supervisor blames individuals.	2.22 (1.10)	2.36 (1.06)	≤0.001**	0.13
4.4 I feel that my supervisor evaluates these situations in a manner that considers the complexity of patient care practices.^R^	2.99 (1.28)	3.23 (1.18)	≤0.001**	0.19
Dimension 5—Institutional support	3.42 (1.04)	3.72 (0.94)	0.329	0.30
5.1 My organization understands that those involved may need help to process and resolve any effects they may have on care providers.^R^	3.45 (1.21)	3.78 (1.11)	≤0.001**	0.28
5.2 My organization offers a variety of resources to help me get over the effects of involvement with these instances.^R^	3.57 (1.23)	3.93 (1.07)	≤0.001**	0.31
5.3 The concept of concern for the well‐being of those involved in these situations is not strong at my organization.	3.25 (1.33)	3.44 (1.31)	0.059	0.14
Dimension 6—Non‐work‐related support	1.66 (0.79)	1.62 (0.77)	≤0.001**	0.05
6.1 I look to close friends and family for emotional support after one of these situations happens.^R^	1.77 (0.94)	1.68 (0.87)	0.301	0.10
6.2 The love from my closest friends and family helps me get over these occurrences.^R^	1.56 (0.78)	1.56 (0.77)	0.987	0
Dimension 7—Professional self‐efficacy	3.48 (0.93)	3.65 (0.89)	≤0.001**	0.19
7.1. Following my involvement I experienced feelings of inadequacy about my patient care abilities.	3.30 (1.16)	3.49 (1.16)	0.020**	0.16
7.2. My experience makes me wonder if I am not really a good healthcare provider.	3.40 (1.31)	3.63 (1.22)	0.027**	0.18
7.3 After my experience, I became afraid to attempt difficult or high‐risk procedures.	3.71 (1.24)	3.96 (1.13)	0.005**	0.21
7.4 These situations do not make me question my professional abilities.^R^	3.49 (1.23)	3.51 (1.21)	0.892	0.02
8—Outcome variable 1—Intention to change jobs	2.85 (1.31)	3.15 (1.30)	≤0.001**	0.23
8.1. My experience with these events has led to a desire to take a position outside of patient care.	2.85 (1.34)	3.17 (1.33)	0.001**	0.24
8.2. Sometimes the stress from being involved with these situations makes me want to quit my job.	2.85 (1.37)	3.13 (1.39)	0.005**	0.20
9—Outcome variable 2—Absenteeism	2.49 (1.28)	2.48 (1.21)	≤0.001**	0.01
9.1. My experience with an adverse patient event or medical error has resulted in me taking a mental health day.	2.51 (1.29)	2.53 (1.28)	0.745	0.01
9.2. I have taken time off after one of these instances occurs.	2.48 (1.31)	2.43 (1.23)	0.841	0.04

Abbreviations: *M*, mean; *SD*, standard deviation. R = the ratings of statements written in negative terms are reversed. **statistically significant Mann–Whitney U Test.

^a^ Cohen’s effect size = 0.2–0.5 small effect, 0.5–0.8 moderate, above 0.8 large.

The most desired support option for both groups was “A respected colleague with whom to discuss the details of what happened” (obstetricians = 4.27/*SD* = 0.88/midwives = 4.50/*SD* = 0.66), although all support options obtained a mean score above 3 points (Table 4). There was also agreement on the least desired option, which was in both cases “The possibility of leaving my unit for a short period of time,” although there was a significant difference in this case between the groups (obstetricians = 3.52/*SD* = 1.41/midwives = 4.04/*SD* = 1.14; *p* ≤ .001).

**TABLE 4:** Desired forms of support

10—desired forms of support	Desired^a^		Not desired^b^		*M* (*SD*)		Mann–Whitney U	Cohen`s effect size^c^
	*N* (%)		*N* (%)					
	Obstetricians	Midwives	Obstetricians	Midwives	Obstetricians	Midwives		
10.1 The ability to immediately take time away from my unit for a little while.	207 (62.7)	305 (78.4)	90 (27.5)	49 (12.6)	3.52 (1.41)	4.04 (1.14)	≤0.001**	0.40
10.2 A specified peaceful location that is available to recover and recompose after one of these types of events.	220 (66.7)	329 (84.6)	68 (20.6)	30 (7.7)	3.75 (1.29)	4.21 (0.96)	≤0.001**	0.40
10.3 A respected peer to discuss the details of what happened.	291 (88.2)	372 (95.6)	17 (4.1)	6 (1.5)	4.27 (0.88)	4.50 (0.66)	0.001**	0.29
10.4 An employee assistance programme that can provide free counselling to employees outside of work.	284 (86.1)	346 (88.9)	20 (6.0)	17 (4.3)	4.25 (0.90)	4.36 (0.83)	0.082	0.01
10.5 A discussion with my manager or supervisor about the incident.	254 (76.9)	293 (75.3)	39 (11.8)	40 (10.3)	4.05 (1.07)	4.05 (1.04)	0.769	0
10.6 The opportunity to schedule a time with a counsellor at my hospital to discuss the event.	272 (82.5)	340 (87.4)	27 (8.2)	25 (6.4)	4.19 (1.00)	4.28 (0.90)	0.414	0.09
10.7 A confidential way to get in touch with someone 24 hours a day to discuss how my experience may be affecting me.	261 (79.1)	327 (84.0)	33 (10.0)	26 (6.7)	4.18 (1.11)	4.26 (0.94)	0.948	0.08

Abbreviations: *M*, mean; *SD*, standard deviation.

^a^ Desired support option = response 4 or 5 points.

^b^ Not desired support option = response 1 or 2 points.

^c^ Cohen’s effect size = 0.2–0.5 small effect, 0.5–0.8 moderate, above 0.8 large.

** statistically significant Mann‐Whitney U Test.

## 4 DISCUSSION

The results of the present study show that more than 60% of Spanish obstetricians and midwives have had feelings of being a second victim, also obtaining high scores on the SVEST‐E questionnaire (above 3). These values were significantly higher among midwives than obstetricians, although the effect size was small.

Some studies that have addressed the second victim phenomenon are in agreement and point out that nurses´ experience more negative feelings than doctors after an adverse event (Harrison et al., 2015; Schrøder, Jørgensen, et al., 2016c). Some researchers postulate that this result could be related to issues of gender, although it is not possible to affirm this with our study data, since the two groups were homogeneous in terms of gender, with the percentage of men and women in both groups (midwives and obstetricians) being very similar. From this perspective, some studies note that negative feelings after, for example a traumatic childbirth, are greater among women (whether they are midwives or obstetricians) (Christoffersen et al., 2020; Schrøder, Jørgensen, et al., 2016b). This has also been pointed out in other studies on second victims (Mira et al., 2015). It must be taken into account that in many countries, the percentage of male midwives is low, if not minimal (Masana et al., 2019). As examples, in the study by Wahlberg et al. (2017), only four of the 1,459 midwives who participated were men, and 11 out of 691 participants were men in the study by Kerkman et al. (2019). Unlike the rest of Europe, Spain has a high proportion of male midwives (Masana et al., 2019). Thus, this possible explanation requires additional research.

Most studies that have investigated the consequences of adverse events such as traumatic childbirth in obstetric professionals have been based on the detection and evaluation of well‐established psychological effects, such as the presence of anxiety, depression, stress, burnout or especially posttraumatic stress disorder (PTSD) (Kerkman et al., 2019; Kruper et al., 2021; Schrøder, Jørgensen, et al., 2016b; Wahlberg et al., 2017). That is why such studies used tools and questionnaires focused mainly on the detection of these psychological problems that, although related to the second victim phenomenon, are not exclusive to it. This has influenced the fact that other important aspects related to the issue of second victims have not been sufficiently studied, such as professional self‐efficacy, work absenteeism or leaving the profession (Burlison et al., 2021).

Therefore, in the present study, a specific measurement instrument was chosen that evaluates the experience of second victims in its multiple spheres, namely the Spanish version of the SVEST (SVEST‐E) (Santana‐Domínguez et al., 2021). The SVEST was the first instrument designed for the study of the second victim phenomenon and serves to obtain reliable information about the adequacy of support resources for second victims (Burlison et al., 2017). Although another questionnaire has also been developed for this purpose—SeViD‐I survey (Strametz et al., 2021)—the SVEST is by far the most used instrument worldwide for the study of the second victim phenomenon, having been translated, adapted and validated in multiple countries and settings (Ajoudani et al., 2021; Brunelli et al., 2018; Chen et al., 2019; Kim et al., 2020; Knudsen et al., 2021; Mok et al., 2020; Scarpis et al., 2021; Winning et al., 2020). The SVEST‐E has also been used recently in Spain to assess the feeling of second victim among cardiology healthcare personnel (Bañeras et al., 2022).

Specifically in the professional obstetric–gynaecological setting, the SVEST has been recently used to evaluate the prevalence of the feeling of second victim among obstetricians, gynaecologists and gynaecological nurses (non‐midwives) in the United States (Finney et al., 2021; Rivera‐Chiauzzi et al., 2021; Torbenson et al., 2021), but no study has focused on establishing possible differences between physicians and nurse–midwives. Although a validation study of the SVEST included midwives in its sample (Knudsen et al., 2021), to the best of our knowledge, the present study is one of the first where this instrument has been used to measure this phenomenon specifically in midwives in the European setting.

The results obtained with the SVEST confirm that the psychological domain is one of the areas most affected when a professional is involved in an adverse event and becomes a second victim (Rivera‐Chiauzzi et al., 2021). However, it also seems to confirm that other areas, sometimes not considered in assessment studies of the second victim phenomenon, are equally affected, such as professional self‐efficacy. In our study, professional self‐efficacy was the third most affected dimension, behind only psychological distress and institutional support, similar to the study by Finney et al. (2021), where this dimension was also the third most affected (in this case behind “non‐work‐related support” and “institutional support”).

Therefore, without ignoring the importance of psychological effects, a comprehensive assessment that pays attention to all the dimensions of this problem should be carried out in the approach to the second victim phenomenon. Recent studies by Torbenson et al. (2021) and Rivera‐Chiauzzi et al. (2021) point in the same direction, indicating that the professional self‐efficacy dimension was at least as affected as the psychological distress dimension after an adverse event, although with lower scores than those obtained in our sample. According to our results, this dimension is more affected in midwives than in obstetricians.

Item 7.3 “After these experiences, I have been afraid to attempt difficult or high‐risk procedures” was the one with the highest score among midwives, also obtaining high scores among obstetricians. Given that obstetric care is complex (Coughlan et al., 2017; Pettker, 2017), this aspect is especially concerning, as it can negatively affect the health care provided to women by professionals who have been involved in an adverse obstetric event (Healy et al., 2016). This is because professionals may want to avoid carrying out a certain risky but necessary procedure at a given time, which can have dire consequences.

The results show higher scores among midwives for 5 of the 9 dimensions included in the SVEST compared with obstetricians. Two dimensions require special emphasis.

On the one hand, midwives had high scores in the Supervisor support dimension, which were also higher than those of their obstetrician colleagues, both in the total score of the dimension and in the four items composing it. According to these results, Spanish midwives perceive receiving little support from their supervisors, noting that this supervision is also performed by midwives. In the reviewed studies, we did not find such high scores in this dimension. For example, in Rivera‐Chiauzzi et al. (2021), this dimension was the one that obtained the lowest score, as in Torbenson et al. (2021) and Finney et al. (2021). In Spain, the perceived support received by midwives involved in an adverse event from their supervisors does not seem adequate. A possible cause could be the lack of empathy on the part of supervising midwives, perhaps linked to their disengagement with direct care. Further research is needed to investigate this aspect. What does seem clear is the need to include topics such as addressing and supporting second victims in the curricular training of supervisors (Torbenson et al., 2021; White & Delacroix, 2020).

On the other hand, given that the score for midwives in the “Intention to change jobs” dimension was 3 points and higher compared with obstetricians (*p* ≤ .001), these scores being higher than those reported by Torbenson et al., 2021, which were below 2.5 points on this dimension.

According to these results, there is a real potential risk that many midwives will seriously consider leaving the profession after being involved in an adverse obstetric event. This consequence of the second victim phenomenon has been previously noted (Burlison et al., 2021; Wahlberg et al., 2017) and is especially worrisome in the particular case of midwives.

In regard to the midwife profession and its work setting, various negative elements have been observed: negative work culture, lack of support for staff, harassment, exhaustion, burnout and leaving the profession (Pezaro et al., 2017). Studies report in certain countries high percentages of midwives who have seriously considered leaving the profession and identify possible causes that provoking this desire as inadequate working conditions, interprofessional conflicts, dissatisfaction with the midwife role played, negative impact on physical and mental health or on family relationships (Harvie et al., 2019; Pugh et al., 2013; Stoll & Gallagher, 2019). Although further studies are required to investigate with greater accuracy the relationship between leaving the profession and second victims, the influence of this phenomenon on the desire to leave the profession should not be underestimated: In our sample, 15.6% of midwives and 19.3% of obstetricians would seriously consider leaving their work after being involved in an adverse event.

It seems clear that for these reasons, it is necessary for health organizations and institutions to design and provide adequate support systems to help professionals involved in this type of situation (Burlison et al., 2021; Finney et al., 2021). This can help mitigate the loss of professionals and promote well‐being among those affected.

In such support systems or resources, according to the results obtained, the option of having a respected peer to discuss the details of what happened is identified as the most desired option, both by midwives and obstetricians. These findings are very similar to those reported in other studies (Burlison et al., 2017; Finney et al., 2021; Mok et al., 2020; Rivera‐Chiauzzi et al., 2021). This support option is known as the “clinician peer support program” (White et al., 2015), and there is already some experience on the effectiveness of this type of support programme in the field of obstetrics (Rivera‐Chiauzzi et al., 2020).

Although there are some proposals for specific support systems for midwives (Christoffersen et al., 2020), an aspect that should be studied in the future is the design of adequate and differentiated support systems according to the professionals involved. The results found in the present study significantly different about some of the desired support options according to the type of professional involved.

Last, this study has some limitations. An obvious limitation is determined by the type of sampling performed; however, professionals from all regions of the country participated in the study in a number that we consider sufficient. In turn, the data collection method used may have influenced the accuracy of the results, since perhaps professionals more familiar with the topic participated in greater number. This issue has also been pointed out in a previous study (Rivera‐Chiauzzi et al., 2021). However, two data points do not support this hypothesis: On the one hand, in both groups, a significant percentage of participants indicated no knowledge of the subject (40.3% for obstetricians and 52.2% for midwives), and on the other hand, a small percentage of participants indicated that the presence of the adverse event that caused the feeling of second victim had occurred less than a year prior (17.6% for obstetricians and 20.6% for midwives). Finally, the survey was conducted at a single point in time, regardless of the time when the triggering adverse event occurred, which could introduce a recall bias, but this limitation is common to studies conducted on this phenomenon (Finney et al., 2021).

## 5 CONCLUSIONS

Although the second victim phenomenon affects all professionals involved in obstetric care, midwives are especially susceptible and sensitive to it.

This phenomenon is multidimensional since it not only implies negative repercussions on the psychological health of affected health professionals but also affects other important aspects, such as absenteeism and leaving the profession, which causes economic and organizational repercussions to health systems. This study provides data that indicate that there are differences in the feeling of second victim according to the type of professional, which has practical implications, since the design of help and support systems should be adjusted to these differences.

## 6 RELEVANCE TO CLINICAL PRACTICE

This is the first study that specifically addresses this phenomenon in the field of obstetric care in Spain and indicates a high prevalence of this problem in both obstetricians and midwives. This finding makes it necessary for health institutions in our country to create adequate policies to mitigate the problems caused by the second victim phenomenon. The creation of support programmes, such as a clinician peer support programme, is proposed as an example of these policies, as well as specialized training of midwife supervisors in this area.

## AUTHOR CONTRIBUTIONS

ISD, HGT, JVS and AMM: Conceptualization and design of the study. ISD, AMM and JJS: Organizational aspects and data collection. HGT, JVS and MBP: Data analysis. ISD, JJS and HGT: Writing—original draft preparation. HGT, JVS and AMM: Review and final editing. All authors read and approved the final manuscript.

## CONFLICTS OF INTEREST

The authors declare that there are no conflicts of interest related to this manuscript.

## Supporting information


Appendix S1

Appendix S2

## Data Availability

Data available on request from the authors.The data that support the findings of this study are available from the corresponding author upon reasonable request.
