# Supplementary material for: Feelings of being a second victim among Spanish midwives and obstetricians
Source: Nurs Open. 2022 May 28;9(5):2356–69. doi: 10.1002/nop2.1249 (PMC9374404; doi:10.1002/nop2.1249)
Supplement: Supplementary file 1 — Appendix S1 [file NOP2-9-2356-s001.docx]

STROBE Statement—Checklist of items that should be included in reports of ***cross-sectional studies***

| ***Title and abstract*** | (*a*) Indicate the study’s design with a commonly used term in the title or the abstract  (*b*) Provide in the abstract an informative and balanced summary of what was done  and what was found | ***Pg 1*** |
| --- | --- | --- |
| ***Introduction*** |  |  |
| ***Background*** | Explain the scientific background and rationale for the investigation being reported | ***3-4*** |
|  | State specific objectives, including any prespecified hypotheses | ***3-4*** |
| ***Methods*** |  |  |
| ***Study design*** | Present key elements of study design early in the paper | ***5*** |
| ***setting*** | Describe the setting, locations, and relevant dates, including periods of recruitment,  exposure, follow-up, and data collection | ***5*** |
| ***Participants*** | (*a*) Give the eligibility criteria, and the sources and methods of selection of  participants | ***5*** |
| ***Variables*** | Clearly define all outcomes, exposures, predictors, potential confounders, and effect modifiers. | ***5-6*** |
| ***Data source*** | For each variable of interest, give sources of data and details of methods of  assessment (measurement). Describe comparability of assessment methods if there is  more than one group | ***6-7*** |
| ***Bias*** | Describe any efforts to address potential sources of bias | ***15*** |
| ***Study size*** | Explain how the study size was arrived at | ***N/a*** |
| ***Quantatitive variables*** | Explain how quantitative variables were handled in the analyses. If applicable,  describe which groupings were chosen and why | ***5-6*** |
| Statistical methods | (*a*) Describe all statistical methods, including those used to control for confounding  (*b*) Describe any methods used to examine subgroups and interactions  (*c*) Explain how missing data were addressed  (*d*) If applicable, describe analytical methods taking account of sampling strategy | ***7-8*** |
| ***Participants*** | a) Report numbers of individuals at each stage of study—eg numbers potentially eligible, examined for eligibility, confirmed eligible, included in the study,  completing follow-up, and analysed  (b) Give reasons for non-participation at each stage  Participants  (c) Consider use of a flow diagram | ***5***  ***N/a*** |
| ***Descriptive data*** | a) Give characteristics of study participants (eg demographic, clinical, social) and  information on exposures and potential confounders  (b) Indicate number of participants with missing data for each variable of interest | ***Pg 8***  ***Table 1*** |
| ***Outcome data*** | Report numbers of outcome events or summary measures | ***8-9*** |
| ***Main results*** | (*a*) Give unadjusted estimates and, if applicable, confounder-adjusted estimates and  their precision (eg, 95% confidence interval). Make clear which confounders were  adjusted for and why they were included  (*b*) Report category boundaries when continuous variables were categorized  Main results 16  (*c*) If relevant, consider translating estimates of relative risk into absolute risk for a meaningful time period | ***8-9*** |
| ***Other analysis*** |  |  |
| ***Discussion*** |  |  |
| ***Key Results*** | Summarise key results with reference to study objectives | ***10*** |
| ***Limitations*** | Discuss limitations of the study, taking into account sources of potential bias or  imprecision. Discuss both direction and magnitude of any potential bias | ***10*** |
| ***Interpretations*** | Give a cautious overall interpretation of results considering objectives, limitations,  multiplicity of analyses, results from similar studies, and other relevant evidence | ***10-15*** |
| ***Generalisability*** | Discuss the generalisability (external validity) of the study results | ***16*** |
| ***Other information*** |  |  |
| ***Funding*** |  | ***This research has no funding*** |
