# Supplementary material for: Feelings of being a second victim among Spanish midwives and obstetricians
Source: Nurs Open. 2022 May 28;9(5):2356–69. doi: 10.1002/nop2.1249 (PMC9374404; doi:10.1002/nop2.1249)
Supplement: Supplementary file 2 — Appendix S2 [file NOP2-9-2356-s002.docx]

Table 1 Checklist for Reporting Results of Internet E-Surveys (CHERRIES)

| ***Item Category*** | ***Checklist Item*** | ***Description*** | ***Page*** |
| --- | --- | --- | --- |
| **Design** | Describe survey design | Major national organisations within obstetrics were contacted and 33 Spanish hospitals were informed about the study and provided with a web link to the questionnaire. | 4 |
| **IRB (Institutional Review Board) approval and informed consent process** | IRB approval | The study was evaluated and approved by the Research Ethics Committee/Drug Research Ethics Committee of Dr. Negrin University Hospital of Gran Canaria (Code n.2020-140-1). | 6 |
|  | Informed consent | When entering the online questionnaire, the first screen showed an informed consent form, clicking on which you accepted it. | 4-5 |
|  | Data protection | We developed and piloted a secure, password-protected web-based data entry portal. Data were anonymised before analysis. |  |
| **Development and pretesting** | Development and testing | A telecommunications engineer tested the online questionnaire. |  |
| **Recruitment process and description of the sample having Access to the questionnaire** | Open survey versus closed survey | This is a closed survey. A secure, password-protected system was tested and provided to participating organisations and supervisors in the participating hospitals.  password-protected system was tested and provided to the organisations and supervisors of participating hospitals. | 4 |
|  | Contact mode | Services were identified and contacted through national and hospice organisations. | 4 |
|  | Advertising the survey | The rationale for the study, ethical approval data protection and data management, investigators, and contacts for further information or concerns. No incentives were offered to complete the study. | 4-5 |
| **Survey administration** | Web/E-mail | The study team developed and tested a secure, password-protected data entry portal.  The study team developed and tested a secure, password-protected data entry portal proposed by one of the research team members. |  |
|  | Context | As indicated above, services were identified and contacted through national organisations and professionals recruited by these associations were encouraged to fill in the SVEST- E |  |
|  | Mandatory/voluntary | It was a voluntary survey as service leads were not mandated to complete it. |  |
|  | Incentives | There was no incentive offered for survey completion. |  |
|  | Time/Date | The data collection period lasted from 15 May to 31 December 2020. | 4 |
|  | Randomization of  items or questionnaires | Items were not randomised or alternated. |  |
|  | Adaptive questioning | The Spanish version of the SVEST was used, which was previously adapted cross-culturally. |  |
|  | Number of Items | 36 items. |  |
|  | Number of screens  (pages) | 2 pages. |  |
|  | Completeness check | The research team audited the data weekly to ensure data entry completeness and sent monthly missing data and incomplete entry reports to the research associates  and administrators, and where consent permitted to relevant respondents to check validity. |  |
|  | Review step | Respondents were able to review and modify their answers before submitting the survey.  Practitioners were provided with a query link infosvest@gmail.com for any queries. |  |
|  | Unique site visitor | A total of 957 professionals completed the questionnaire with an average visit time of 7:05 minutes. |  |
|  | View rate (Ratio of unique survey visitors/unique site  visitors) | Unique users accounted for 719 of the total 957 in a bounce rate of 14.75%. |  |
|  | Participation rate (Ratio of unique visitors who agreed to participate/unique first survey page visitors) | The participation rate was 75.13%. |  |
|  | Completion rate (Ratio of users who finished the survey/users who agreed to participate) | The completion rate was 94.31%. |  |
| **Preventing multiple entries from the same individual** | Cookies used | To maximise reliability, guaranteeing the anonymity of users were cookies; for this purpose, the Google Analytics tool was used. |  |
|  | IP check | IP check was not carried out. |  |
|  | Log file analysis | Participation was anonymous, so no registration was used. |  |
|  | Registration | It was not used. |  |
| **Analysis** | Handling of incomplete  questionnaires | All available data were analysed.  Missing data were not imputed. |  |
|  | Questionnaires submitted with an atypical timestamp | Not applicable. |  |
|  | Statistical correction | Not applicable. |  |
